# Comparative evaluation of the utility of two oral examination tools in assessing oral health in stroke patients with indwelling gastric tubes

**DOI:** 10.1371/journal.pone.0325688

**Published:** 2025-06-09

**Authors:** Shuangyan Tu, Menglin Jiang, Rong Yang, Zhiqiang Deng, Cairong Zhu, Muke Zhou, Zhangyan Peng, Lihong Zhao

**Affiliations:** 1 Department of Neurology, West China Hospital, Sichuan University/West China School of Nursing, Sichuan University, Chengdu, Sichuan, China; 2 West China Xiamen Hospital of Sichuan University, Amoy, Fujian, China; 3 Department of Epidemiology and Health Statistics, West China School of Public Health and West China Fourth Hospital, Sichuan University, Chengdu, Sichuan, China; 4 Department of Neurology, West China Hospital, Sichuan University, Chengdu, Sichuan, China; 5 Department of Neurology, West China School of Public Health and West China Fourth Hospital, Sichuan University, Chengdu, Sichuan, China; 6 Department of Radiology, West China Hospital, Sichuan University/West China School of Nursing, Sichuan University, Chengdu, Sichuan, China; Universidade dos Açores Departamento de Biologia: Universidade dos Acores Departamento de Biologia, PORTUGAL

## Abstract

**Purpose:**

To identify the scale that is more suitable for oral health assessment in stroke patient population with indwelling gastric tubes.

**Methods:**

A total of 198 patients with indent gastric tubes were selected from 1250 stroke patients to evaluate their oral health using both the BOAS and the OHAT scales. The scores obtained from both scales were then compared to evaluate the feasibility, reliability, and validity of each scale in assessing oral health among stroke patients with indwelling gastric tubes.

**Results:**

The results showed that both the BOAS and OHAT scales exhibited good reliability and validity in stroke patients with indwelling gastric tubes. The Cronbach’s alpha coefficients of BOAS and OHAT in stroke patients with indwelling gastric tubes were 0.89 and 0.91, respectively. In the exploratory factor analysis, one and two common factors were extracted from the two scales, with cumulative variance contributions of 65.89% and 71.85%, respectively. In addition, potential influencing factor correlation analysis found that gender and marital status had a significant correlation with the BOAS score(P < 0.05), the Activities of daily living (ADL) score was found to be significantly correlated with the OHAT score (P < 0.05). Drinking, smoking, income, consciousness, and the result of the water swallow test were all correlated with BOAS and OHAT scores(P < 0.01).

**Conclusions:**

The BOAS and OHAT have demonstrated good reliability and validity and in their ability to assess the oral health of stroke patients with indwelling gastric tubes. Therefore, it is recommended that the selection of oral assessment scales should be further refined in different disease stages of stroke patients to assess the oral health status of patients more accurately and personalized.

## Introduction

Stroke continues to be the leading cause of both mortality and morbidity in China, as evidenced by data from the Global Burden of Disease Study which shows a consistent increase in stroke incidence rates [1]. A significant portion of stroke patients, ranging from 70% to 80%, experience varying degrees of disability is a stark reminder of the devastating consequences of this disease. Paralysis, swallowing difficulties, depression, anxiety, and an overall rise in disease burden [2].

Oral health is defined by the World Health Organization (WHO) as a state of being free from mouth and facial pain, oral and throat cancer, oral sores, birth defects such as cleft lip and palate, periodontal disease, tooth decay and tooth loss, and other diseases and disorders that affect the oral cavity [3]. Studies had shown that there is a positive correlation between oral health and the risk of stroke. Therefore, a comprehensive oral hygiene protocol is essential for enhancing oral health in inpatient stroke rehabilitation patients. Stroke patients often experience dysfunction in the tongue, pharynx, palate, and masticatory muscles due to nerve damage. This results in varying degrees of swallowing difficulties [4]. The incidence of swallowing dysfunction is 22% −65% [5]. In clinical practice, the swallowing function of patients with dysphagia was evaluated, and the indwelling gastric tube was placed for patients with severe dysphagia and unable to eat by mouth. In addition, some severe stroke patients or stroke patients with varying degrees of disturbance of consciousness, to ensure nutrition and fluid supply, nasogastric feeding via an indwelling gastric tube is often initiated within the first seven days of onset. Previous studies have found that the indentation of gastric tubes in stroke patients can reduce the salivation, which is an important natural defense mechanism against oral bacteria. A reduced salivary flow can change the oral environment, making it more conducive for the proliferation of microorganisms such as dental plaque, lead to the occurrence of stroke-related pneumonia, prolong the hospital stay of patients, and the economic and family burden is far greater than that of ordinary stroke patients [6].

It is crucial to manage oral health for stroke. Oral health assessment is the foundation of oral health management, playing an important role in assessing oral health risks, identifying oral health issues, and timely diagnosing and treating oral diseases [7]. It can effectively guide oral health management and improve patient quality of life. However, there is still a lack of unified and standardized oral assessment tools in China, especially for patients with indwelling gastric tubes. The Modified Beck Oral Assessment Scale (BOAS) and the Oral Health Assessment Tool (OHAT) have been proven to be reliable and effective in assessing oral health in ICU patients and stroke patients, respectively [8]. The Oral Health Assessment Tool (OHAT) is an oral assessment tool recommended by the Canadian Stroke Guidelines. It has a relatively wide range of applications and is often used by nurses [9]. In this study, we hypothesized that both BOAS and OHAT scales were suitable for the oral health assessment of stroke patients with indent gastric tube, but the emphasis of the two oral assessment scales was different. Therefore, the aims of the study were to assess the oral health of patients with cerebral apoplexy indwelling gastric tubes by using BOAS and OHAT scales and compare their applicability to explore a more suitable oral assessment scale for stroke patients with cerebral apoplexy indwelling gastric tubes.

## Materials and methods

### Patients

**Fig 1:**
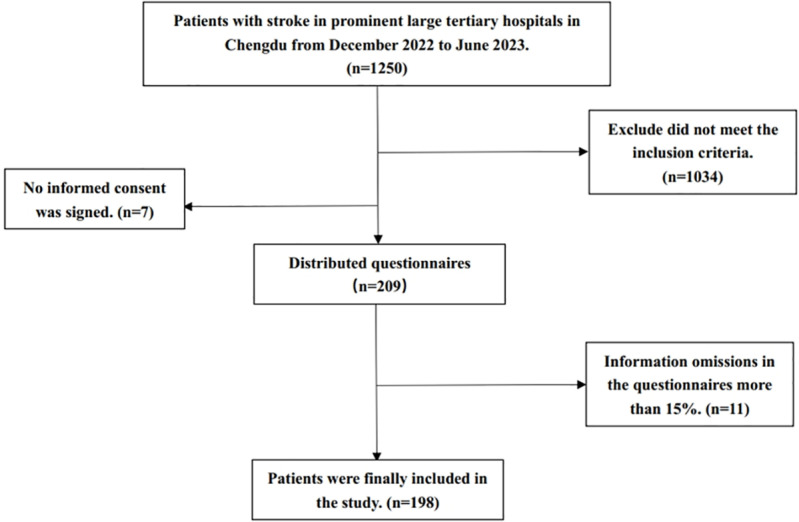
Flow diagram showing the process of participant selection and exclusion.

From December 2022 to June 2023, purposive sampling was employed to select patients with stroke who also had swallowing disorders and indwelling gastric tubes as the subjects of study in prominent large tertiary hospitals in Chengdu. These hospitals are recognized as national centers for diagnosing and treating complex and critical cases in western China, with an average daily treatment capacity of approximately 17,000 individuals. Furthermore, their medical resources extend throughout the entire western region of China. The inclusion criteria were as follows:(1) Patients whose primary diagnosis was subarachnoid hemorrhage, intracerebral hemorrhage, or cerebral infarction (International Classification of Diseases, 10th revision (ICD-10) [10]: I60x, I61x, I63x, I67x); (2) Patients with retained gastric tubes; (3) Patients who are informed and agree to participate in the study. Exclusion criteria:(1) Patients with combined malignant tumors and severe heart, liver, and kidney disorders. (2) Patients with oral diseases or oral trauma. A total of 1250 stroke patients were screened during the study, among which 198 patients with dysphagia required gastric tube indentation (Fig 1). The general information of the Patients is shown in Table 1. This research has been approved by the Medical Research Council, Scientific and Research Committee of the hospital (No: 20221385). All patients provided written consent for participation. The study followed the recommendations of STROBE (Strengthening the Reporting of Observational Studies in Epidemiology).

**Table 1:** Participant characteristics(n = 198).

Parameter		n (%)
**Sex (%)**	Female	54 (27%)
	Male	144 (73%)
**Age (%)**	≤44	13 (7%)
	45-64	60 (30%)
	≥65	125 (63%)
**Marital status (%)**	Single	19 (10%)
	Married	179 (90%)
**Location (%)**	Rural	62 (31%)
	Town	136 (69%)
**Income (%)**	≤2000	42 (21%)
	2001-4000	114 (58%)
	4001-6000	33 (16%)
	≥6001	9 (5%)
**Education (%)**	Illiteracy	11 (5%)
	Primary education	45 (23%)
	secondary education (Junior high school, senior high school and technical secondary school)	93 (47%)
	higher education (Undergraduate and above)	49 (25%)
**Smoking (%)**	No	125 (63%)
	Yes	73 (37%)
**Drinking (%)**	No	135 (68%)
	Yes	63 (32%)
**Consciousness (%)**	Awake	108 (54%)
	somnolence	50 (25%)
	Stupor	24 (12%)
	Comatose	10 (5%)
	Confusion	1 (1%)
	Delirious	5 (3%)
**Water Swallow Test (%)**	1	18 (9%)
	2	18 (9%)
	3	33 (17%)
	4	18 (9%)
	5	111 (56%)

### Instrument

**The Modified Beck Oral Assessment Scale (BOAS):** There are five components of BOAS [8]: lips, oral mucosa and gums, tongue, teeth, and saliva. Each item is assigned a score from 1 to 4 points, with a total possible score ranging from 5 (best) to 20 (worst). The higher the BOAS score, the more severe the damage to oral function. The normal values for the dimensions of lips, oral mucosa, gums, tongue, teeth, and saliva are 1.5 points indicate good oral function; 6–10 points indicate mild functional impairment; A score of 11–15 indicates moderate functional impairment; A score of 16–20 indicates severe functional impairment.

**The Oral Health Assessment Tool (OHAT):** The Oral Health Assessment Tool (OHAT) is developed based on the BOHSE scale [9]. The scale includes eight items: lips, tongue, gums, saliva, natural teeth, dentures, oral hygiene, and toothache. Each item adopts a 3-level scoring method, with a single score of 0–2. The total score for all eight items ranges from 0 (best oral health) to 16 (worst oral health). The lower the score, the better the oral health condition, and vice versa.

**The Barthel Index:** The Barthel Index [11] is a measurement tool designed and developed in 1965 to evaluate the Activities of daily living (ADL). A series of daily independent behaviors of patients were measured using ten items, including eating, bathing, grooming, and dressing. The total score ranges from 0 (complete dependence in all activities) to 100 (full independence in all activities). A lower score indicates that the patient is less self-reliant and more dependent.

### Data collection

On the premise that patients and their families fully understand the purpose and significance of this study, with the informed consent of patients, a questionnaire survey was conducted on 198 stroke patients with indwelling gastric tubes using the BOAS and OHAT, respectively. Assessors need to undergo professional training and be qualified. In this study, stroke patients were assessed by professionals using the BOAS and OHAT scales. To protect patient privacy and minimize potential deviations in the results, one-on-one individual evaluations were arranged. This cross-sectional study requires a sample size 5–10 times the number of items. With 21 items, the initial sample size should be 105–210. Considering 10%−20% attrition rate, the final required sample size is 116–231.A total of 209 oral health assessment questionnaires were distributed in this study. After excluding invalid questionnaires, 198 valid questionnaires were collected, with an effective recovery rate of 94.74%.

### Evaluating indicator

Cronbach α coefficient was used to measure the reliability of the two scales. The reliability of the scale was divided into external reliability and internal reliability, among which internal reliability, also known as internal consistency, reflects the degree of correlation between various dimensions. Cronbach α coefficient is the most commonly used index to measure internal reliability. The larger the Cronbach α coefficient is, the higher its internal reliability is. It is generally believed that when n > 100, α > 0.75 is a high reliability scale. Validity refers to the degree to which the scale used can measure the characteristics that the researcher intends to measure. Structural validity is the examination of whether the scale used can reflect the assumed theoretical structure based on actual measured data. The higher the structural validity, the more it can truly reflect the structure of the theory or the characteristics to be measured [12]. The Bland-Altman plot was used to compare the relationship between the two scales, and 95% of the scatter points in the Bland-Altman plot were within the consistency limit of the difference between the two scales (95% confidence interval Mean±1.96SD of the difference between the two scales), so it was considered that the two scales could be used interchangeably.

### Data analysis

All data input SPSS26.0 statistical analysis software, using double input to ensure the accuracy of data input process. Quantitative data was described by mean ± standard deviation, while Qualitative data was statistically described using examples and percentages. Cronbachα coefficient was used to evaluate the internal consistency reliability of the two scales, and factor analysis was used to evaluate the structural validity of the two scales. The correlation between two scales was analyzed using Pearson correlation analysis, and the relationship between the two scales was compared using the Bland Altman plot. Spearman correlation analysis was used to evaluate the correlation between potential influencing factors and oral health scores of stroke patients with indentured gastric tube. A P-value of less than 0.05 (*P* < 0.05) was considered to be statistically significant.

## Results

### Reliability and validity analysis of the oral evaluation scale for stroke patients with indwelling gastric tubes

The Cronbach’s alpha coefficient of BOAS for stroke patients with indwelling gastric tubes was 0.89, p < 0.05, and Cronbach’s alpha coefficient of OHAT was 0.91, p < 0.05. See Table 2. KMO and Barrett’s sphericity tests were performed on the data, and the KMO values of the two scales were 0.859 and 0.873, respectively, with P < 0.01, indicating that the two scales are suitable for exploratory factor analysis. Using principal component analysis, based on the criterion of eigenvalues>1,BOAS scale extracted one common factor (lips), OHAT scale extracted two common factors (lips and tongue), with cumulative variance contribution rates of 65.89% and 71.85% for BOAS and OHAT, respectively. See Table 3.

**Table 2:** Reliability of BOAS and OHAT.

Scale	Cronbach α	Standard Cronbach α	F	*p*
BOAS	0.88	0.89	13.88	<0.05
OHAT	0.91	0.91	26.01	<0.05

**Table 3:** Component matrix of oral health score (n = 198).

BOAS		OHAT		
Item	Factor 1: Lips	Item	Factor1: Lips	Factor2: Tongue
Lips	0.85	Lips	0.85	0.16
Gingiva and oral mucosa	0.85	Tongue	0.87	0.21
Tongue	0.87	Gingiva	0.83	0.19
Teeth	0.77	Saliva	0.77	0.10
Saliva	0.72	Nature teeth	0.61	0.60
Eigenvalues	3.30	Dentures	0.60	0.58
cumulative variance contribution rate	65.89%	Quality of tooth hygiene	0.79	0.14
		Teeth pain	−0.01	0.91
		Eigenvalues	4.09	1.65
		cumulative variance contribution rate	51.17%	71.85%

### Comparison of two evaluation scales

**Fig 2:**
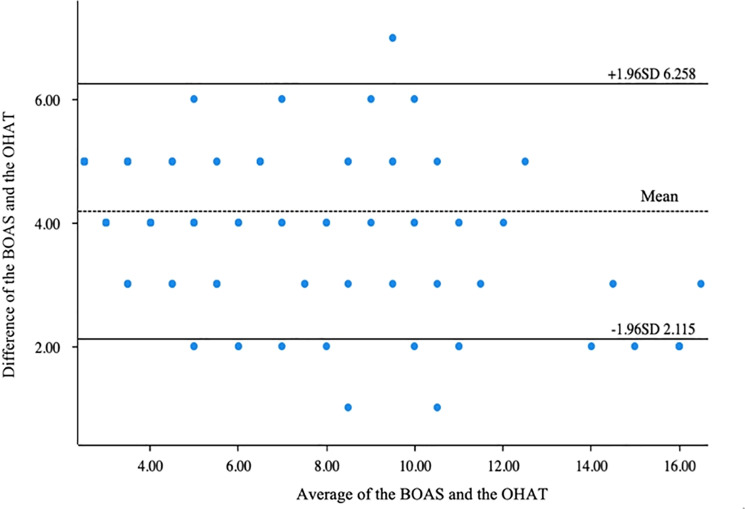
Bland-Altman Plot of the BOAS and the OHAT.

There was a significant correlation between the oral health scores of stroke patients with indwelling gastric tubes assessed by BOAS and OHAT (Pearson correlation coefficient r = 0.93, P < 0.01). See Table 4. The mean value of the score difference between the two scales was 4.187, and the standard deviation of the difference was 1.057. Therefore, the limits of agreement of the two scales were (2.115, 6.259). The Bland-Altman plot was used to compare BOAS and OHAT, and more than 95% of the scattered points were within the limits of agreement (Fig 2).

**Table 4:** The correlation analysis results between potential influencing factors and oral scores.

	1	2	3	4	5	6	7	8	9	10	11	12	13
BOAS (1)	1												
OHAT (2)	0.93**	1											
Sex(3)	0.14*	0.12	1										
Age(4)	0.07	0.07	−0.20**	1									
Marriage(5)	0.14*	0.12	0.15*	−0.14*	1								
Location(6)	0.02	0.06	−0.02	0.29**	0.01	1							
Drinking(7)	0.22**	0.16*	0.37**	−0.09	0.11	−0.03	1						
The Barthel Index (8)	−0.13	−0.16*	0.14	−0.04	−0.06	0.10	0.07	1					
WST (9)	0.24**	0.27**	−0.05	0.01	0.10	−0.01	−0.08	−0.39**	1				
Consciousness(10)	−0.33**	−0.34**	−0.03	0.19**	−0.02	−0.03	−0.05	0.30**	−0.37**	1			
Income(11)	0.29**	0.30**	0.10	−0.12*	−0.04	−0.23**	−0.04	−0.08	0.08	−0.18*	1		
Education(12)	0.09	0.09	−0.05	0.06	−0.04	0.06	−0.14*	0.07	−0.03	0.02	0.15*	1	
Smoking(13)	0.28**	0.23**	0.44**	−0.10	−0.04	−0.07	0.62**	0.02	0.01	−0.09	0.03	−0.10	1

* p < 0.05 ** p < 0.01.

### The correlation between potential influencing factors and different oral scores

Correlation analysis between potential influencing factors such as gender, age, marriage, income, education, location, drinking, smoking, water swallow test (WST) and consciousness status of patients with both BOAS and OHAT scores. See Table 4. The results showed that gender and marriage were only correlated with the BOAS score (P < 0.05) and not with the OHAT score. The Barthel Index was only related to the OHAT score (P < 0.05) and not the BOAS score. Drinking, smoking, income, consciousness, and the result of the water swallow test were all correlated with BOAS and OHAT scores(P < 0.01). Age, location, and education were not associated with the BOAS scores and the OHAT scores(P > 0.05).

## Discussion

### Both scales demonstrate good reliability and validity

The evaluation provided here demonstrates that the BOAS and OHAT scales are both reliable and valid tools for assessing oral health in stroke patients with indwelling gastric tubes. Cronbach’s alpha coefficient was 0.89 for BOAS and 0.91 for OHAT, indicating good internal consistency confidence for oral health assessment of stroke patients with indwelling gastric tubes. Haghighi et al. [13] measured an internal consistency α coefficient of 0.92 for BOAS in critically ill patients with ventilator intubation, which was slightly higher than 0.89 for stroke patients with indwelling gastric tubes in this study. Finotto et al. [14] used the OHAT to evaluate the oral health of the elderly with cognitive impairment and calculated the Cronbach’s alpha coefficient of the OHAT scale to be 0.82, slightly lower than in this study. This result may suggest that OHAT may have limitations for stroke patients with indwelling gastric tubes in this study. In this study, some patients were unable to eat autonomously due to consciousness disorders, tracheal intubation, and other reasons and could only receive nutritional support through indwelling gastric tubes. These patients could not express themselves normally due to the presence of wounds in the tracheal intubation or the influence of consciousness disorders, which may affect the subjective evaluation results of “toothache” in OHAT, thereby affecting the accuracy of the evaluation results.

In this study, the structural validity of the scale was evaluated by exploratory factor analysis. BOAS and OHAT extracted 1 and 2 common factors, respectively. The factor loading was greater than 0.4, and the cumulative variance contribution rate was 65.89% and 71.85%, respectively, higher than the general standard (>50%), indicating that each item contributed more to the scale. The scale has good structural validity, and the results are similar to those of Chalmers et al. and Jahanshir et al. [9,15]. Due to the lack of a gold standard for evaluating the oral health of stroke patients in clinical practice, this study did not conduct a criterion-related validity test for the oral health score of stroke patients with indwelling gastric tubes.

### The assessment methods of the two scales were consistent

This study compared two observational assessment tools commonly used to assess oral health in critically ill patients, and the results showed a strong correlation between BOAS and OHAT and the Bland-Altman Plot showed that the two could play an alternate role in the assessment of oral health in stroke patients with indwelling gastric tubes. BOAS and Mucosal Plaque Score were compared by Haghighi et al. [13] for patients undergoing ventilator intubation, and a correlation coefficient of 0.92 was found. However, Cohen’s [16] point was well-taken in that a comprehensive evaluation of oral health should encompass more than just local features of the oral cavity. Thus, the mucous membrane and plaque are considered only as part of the mucoplaque Score, which may not provide an accurate assessment of oral health. In this study, the strong correlation between BOAS and OHAT, with a coefficient of 0.93, suggested that both tools are capturing similar aspects of oral health. Both BOAS and OHAT include assessments of mouth, teeth, tongue, saliva, etc., and the similarity of item contents may be related to the results of the strong correlation between the two scales.

### Correlation between potential influencing factors and different oral health scores

It is noteworthy that although the two scales in this study can play an interchangeable role, the correlation analysis between the potential factors and the scores of the two scales in this study found that gender and marriage were only related to BOAS scores, while ADL scores were only related to OHAT scores. Demographic factors such as gender and marriage can indirectly infer the oral health behavior of patients. Research showed that women may have better oral hygiene and health habits than men [17]. According to research on attachment theory [18], the relationship between couples provides emotional support, and they pay more attention to the appearance of their mouth and face. At the same time, mutual care could timely detect and correct behaviors that affect their health. Therefore, in this study, gender and marriage were only correlated with BOAS score, but not with OHAT, suggesting that BOAS may be a better predictor of the impact of demographic factors on oral health, and can be used as a tool for pre-hospital oral assessment of stroke patients with indentation gastric tube. The ADL scores mirror the self-care capacity and quality of life of patients. Research has demonstrated that patients with higher ADL scores and better self-care abilities are less prone to suffer from mental health issues post-stroke and can return to work sooner. In this study, the Barthel Index was merely associated with OHAT, indicating that OHAT might be more appropriate for the oral health assessment of stroke patients with indwelling gastric tubes during the rehabilitation phase and after discharge. For stroke patients, oral health management should be a continuous and comprehensive process using corresponding oral health assessment tools at different stages of disease progression can enable more accurate assessment [19].

In our study, smoking and drinking were associated with BOAS and OHAT scores. Studies have shown that smoking and drinking alcohol are risk factors for oral health problems [20]. Harmful substances in cigarettes and alcohol can have a direct impact on the gum microcirculation, damaging the oral mucosa epithelium, and reduce oral health. Stroke was a high-risk group of oral health problems in smoking patients [21]. When combined with smoking and drinking, these factors can create a perfect storm for oral health problems. There was a correlation between income and oral health of stroke patients with indwelling gastric tubes. The heavy medical burden of stroke can make it difficult for low-income patients to prioritize oral health care, which can lead to worse oral health outcomes [22]. In addition, the level of consciousness and the grading of the water swallow test were all related to the scores of BOAS and OHAT. For patients with a water swallow test greater than level 3, who were in a coma or had consciousness disorders, their chewing and swallowing functions weakened or even disappeared, causing them to be unable to eat through the mouth. The patient’s oral saliva secretion decreases, and the oral mucosa cannot be fully moisturized, leading to dry mouth syndrome and oral health problems [23,24]. Therefore, for patients with swallowing dysfunction and coma, it is more important to strengthen oral care and pay attention to their oral health.

This study found that age was not correlated with BOAS and OHAT scores, which was different from the results of Meaghan et al. [17] This might be associated with the trend of younger onset of stroke in recent years. According to statistics, youth stroke accounts for about 15% to 18% of all strokes, and the proportion of severe cases is relatively high [25]. As the severity of severe stroke increases, oral health is increasingly being neglected. This result deserves further consideration. For patients suffering from ischemic stroke, thrombolysis represents the preferred therapeutic option during the extremely early stage (within 4.5 hours after onset), and gingival bleeding frequently indicates the occurrence of thrombolysis-related complications and has an impact on the thrombolysis process [26]. Studies have shown that the oral health rate of older people over 65 is less than 15%, and the elderly are mostly accompanied by dental caries, gingival bleeding, and other oral problems [27]. How to discriminate between the gingival bleeding resulting from oral health issues and that caused by thrombolysis merits further exploration.

## Conclusions

The BOAS and OHAT scales have good reliability and validity and are effective assessment tools for screening oral health in stroke patients with indwelling gastric tubes. Refining the oral assessment through a personalized approach is crucial for more effectively evaluating the oral health status of stroke patients at various stages of the disease. By adopting this approach, we can significantly lessen the disease burden on patients, leading to improved rehabilitation outcomes and a better quality of life. This personalized strategy is crucial for the well-being of stroke survivors.

## Limitations

The study has several limitations. First, the subjects were only from the National Critical Care Center in western China, which limits the generalizability of our findings. In future studies, the sample size and distribution should be expanded to validate the applicability of both oral health assessment tools. In addition, this study only investigated the oral health level of stroke patients with indwelling gastric tube during hospitalization, but oral health is dynamically changing. In future studies, BOAS and OHAT scales can be used to continue to follow up the oral health level of stroke patients with indwelling gastric tube after discharge, and further explore the relationship between the two oral health assessment tools and different stages of stroke.
